# Antarctic Marine-Derived Fungi: Metabolomic Signatures and Antibiofilm-Driven Anti-Infective Potential Against Drug Resistant Pathogens

**DOI:** 10.3390/md24080267

**Published:** 2026-08-02

**Authors:** İbrahim S. Uras, Pedro H. S. Candido, Catarina M. Luís, Vanda Marques, Cecília M. P. Rodrigues, Rita G. Sobral, Anelize Baurmeister, Belma Konuklugil, Susana P. Gaudêncio

**Affiliations:** 1 Faculty of Pharmacy, Department of Pharmacognosy, Ağrı İbrahim Çeçen University, 04100 Ağrı, Turkey; ibrahimseydauras@hotmail.com; 2 Department of Fundamental Chemistry, Institute of Chemistry, University of São Paulo, São Paulo 05508-000, SP, Brazil; pedrovip21@usp.br (P.H.S.C.); anelize@iq.usp.br (A.B.); 3 Associate Laboratory i4HB, Institute for Health and Bioeconomy, NOVA School of Science and Technology, NOVA University of Lisbon, Campus Caparica, 2819-516 Caparica, Portugal; cma.luis@campus.fct.unl.pt (C.M.L.); rgs@fct.unl.pt (R.G.S.); 4 UCIBIO—Applied Molecular Biosciences Unit, Chemistry and Life Sciences Departments, NOVA School of Science and Technology, NOVA University of Lisbon, Campus Caparica, 2819-516 Caparica, Portugal; 5 Research Institute for Medicines (iMed.ULisboa), Faculty of Pharmacy, Universidade de Lisboa, Av. Professor Gama Pinto, 1649-003 Lisboa, Portugal; vanda.marques0206@gmail.com (V.M.); cmprodrigues@ff.ulisboa.pt (C.M.P.R.); 6 Faculty of Pharmacy, Department of Pharmacognosy, Lokman Hekim University, 06560 Ankara, Turkey; belma.konuklugil@gmail.com

**Keywords:** extreme environments, marine-derived Antarctic fungi, natural products, secondary metabolites, untargeted LC–MS/MS, GNPS molecular networking, antimicrobial, antibiofilm and cytotoxicity activities, blue biotechnology

## Abstract

Antarctic marine-derived fungi represent an underexplored reservoir of bioactive secondary metabolites shaped by extreme environmental pressures. In this study, nine fungal isolates obtained from Antarctic macroalgae, lichens, sponge tissue, and sediments were evaluated for their antimicrobial, anticancer, antibiofilm, and metabolomic profiles. Untargeted LC–MS/MS molecular networking (GNPS) revealed a chemically rich metabolome, dominated by alkaloids, followed by polyketides, meroterpenoids, and diketopiperazines, with *Penicillium crustosum* (A15A) emerging as a major biosynthetic contributor. The annotation of structurally diverse metabolites, including roquefortines, viridicatin derivatives, andrastins, and multiple diketopiperazines, highlights the metabolic plasticity of Antarctic fungi. Anticancer evaluation indicated cytotoxicity, with *Aspergillus awamori* (A30), *Alternaria malorum* (A36), and *Cladosporium malorum* (A38) displaying activity toward HCT 116 colorectal cancer cells at IC_50_ ≥ 30 µg/mL. Extracts were screened against methicillin-resistant *Staphylococcus aureus* (MRSA, COL), methicillin-susceptible *S. aureus* (MSSA, NCTC8325 4), and *Escherichia coli* K12, revealing low to no activity against these pathogens. Six of the nine isolates exhibited strong antibiofilm activity without inhibiting planktonic bacterial growth, indicating selective biofilm inhibition against MSSA and meeting the criteria for clinical developmental “hits”. Biofilm inhibition ranged from 81.10% to 98.50%, with A15A showing the highest activity (98.50% at 250 µg/mL), followed by A36 (91.16% at 31.35 µg/mL). *Botrytis* sp. (A22A), *P. chrysogenum* (A7), *Ulocladium microsporum* (A24B), and A30 also demonstrated strong antibiofilm activity (81.10–85.89%). To our knowledge, this is the first report describing antibiofilm activity of Antarctic fungal extracts.

## 1. Introduction

Antarctica, the coldest and most inhospitable continent on Earth, imposes extreme selective pressures on its resident biota. To survive in this harsh environment, organisms must withstand persistent sub-zero temperatures, intense UV radiation, and severe nutrient limitation. Many species, particularly microorganisms, have evolved remarkable physiological and biochemical adaptations producing secondary metabolites with unique chemical structures and bioactivities, which often differ substantially from those produced by related taxa in temperate environments [1].

Microbial infections and the escalating resistance of microorganisms to existing drugs represent major global challenges for both public and environmental health. A key mechanism through which microorganisms acquire and maintain resistance is the formation of biofilms. Biofilm development not only enhances tolerance to antimicrobial agents but also contributes to persistent and recurrent infections, spoilage in processed foods, and the colonization and malfunction of medical devices [2]. These biofilm-associated challenges highlight the urgent need for new strategies and metabolites capable of disrupting or preventing biofilm formation.

Biofilm formation is a widespread trait among bacteria; pathogens embedded within these structured communities exhibit markedly increased resilience to current treatment options. The biofilm matrix acts as a physical and chemical barrier, reducing antibiotic penetration and shielding cells from host immune responses. As a result, even bacteria without inherent genetic resistance can withstand antimicrobial therapies simply by residing within a protective biofilm layer, making their eradication significantly more difficult [3]. Natural products with antibiofilm activity typically act through three principal mechanisms: inhibiting biofilm formation, dispersing mature biofilms, and impairing biofilm-embedded cells. Together, these complementary modes of action provide a strategic framework for developing next-generation therapeutics targeting biofilm-associated infections [4].

A wide range of metabolites with antibiofilm activity has been isolated from diverse natural sources, including plants, microorganisms, and marine invertebrates [5,6]. Examples include ellagic acid glycosides, hamamelitannin, carolactone, phenazines, meridianin D, napyradiomycins, and marinone derivatives, such as madeirone and neomarinone [7,8,9,10]. Antibiofilm agents originating from Antarctic organisms include pentadecanal, isolated from the Antarctic bacterium *Pseudoalteromonas haloplanktis* TAC125, which inhibited >50% of *Staphylococcus epidermidis* biofilm formation at 60 µg/mL [11]. Another example is darwinolide, a diterpene isolated from the Antarctic sponge *Dendrilla membranosa* Pallas, which exhibits selective activity against biofilm-associated infections caused by methicillin-resistant *Staphylococcus aureus* (MRSA), reducing pre-formed MRSA biofilms by 50% at 33.2 µM [12].

Within this context, evaluating whether marine-derived fungal isolates exhibit antibiofilm activity at the crude extract level represents a critical first step. Although fungal secondary metabolites with antimicrobial activity are well documented, their antibiofilm potential remains comparatively understudied, and seaweed-associated endophytic fungi, in particular, represent a poorly explored reservoir of chemically diverse metabolites [13]. To our knowledge, there are no reports of antibiofilm metabolites from Antarctic fungi.

In this study, fungal isolates obtained from Antarctic macroalgae, lichens, sponge tissue, and marine sediments were evaluated for their antimicrobial, anticancer, and antibiofilm activities. Additionally, metabolomic fingerprinting using high-resolution LC–MS/MS-based untargeted metabolomics and molecular networking were performed. The significance of this work is strengthened by the uniquely Antarctic origin of the samples, as several of the examined species, and their corresponding extracts, have never been investigated for these biological activities.

## 2. Results and Discussion

### 2.1. Antarctic Fungi Isolates

**Figure 1:**
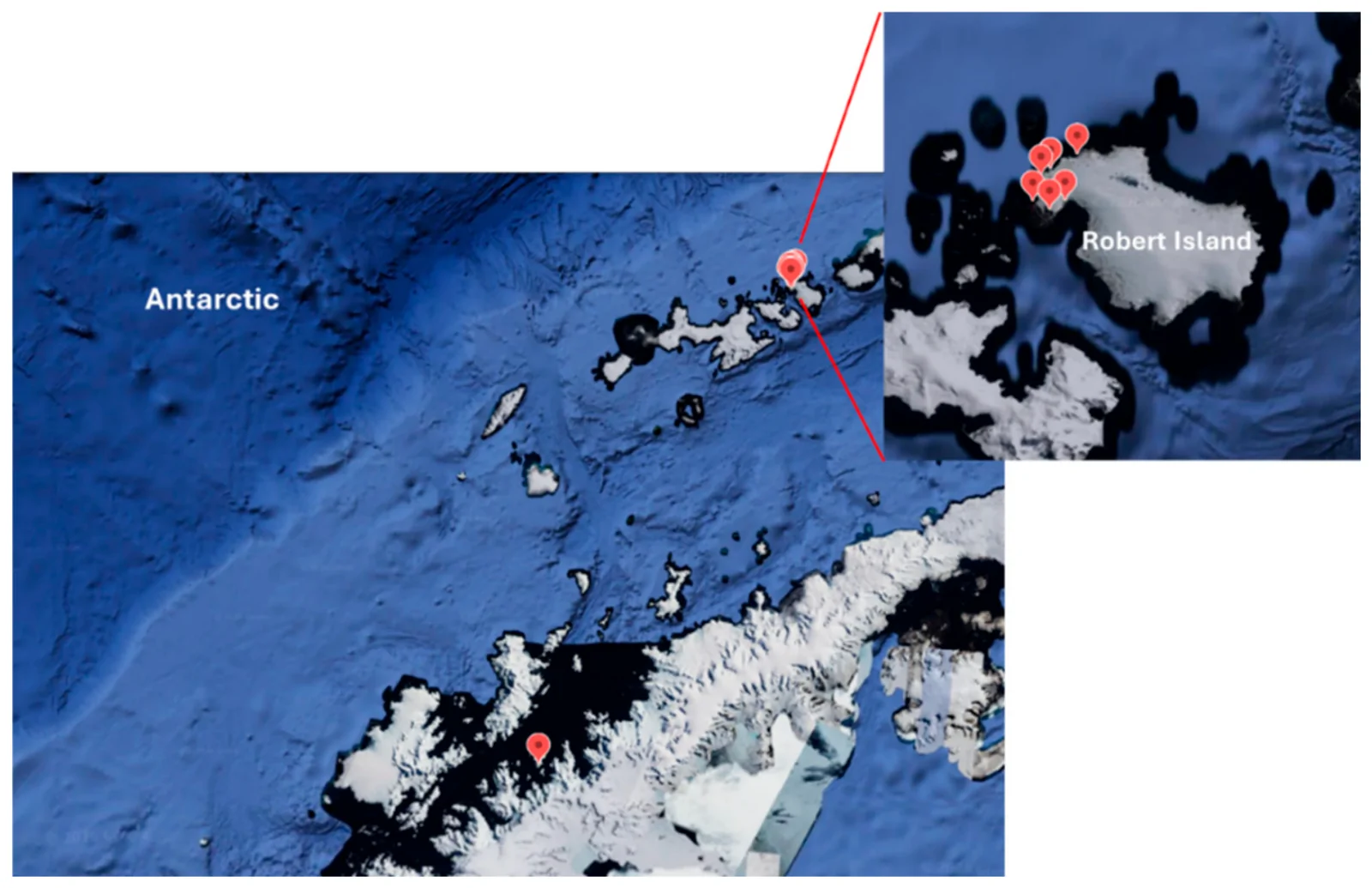
Geographic location of eight sampling sites on Robert Island in the South Shetland Archipelago, and Graham coast of the continental Antarctic Peninsula. The inset highlights the distribution of collection points (red markers) within Robert Island, linked to the broader Antarctic region. This map was retrieved from Google Earth (https://earth.google.com/web/, accessed on 21 May 2026).

Nine Antarctic marine-derived fungal isolates (A7, A15A, A21F, A22A, A24B, A29A, A30, A36, and A38) were obtained from diverse marine substrates, representing a taxonomically and ecologically heterogeneous group of filamentous fungi adapted to the extreme conditions of the Southern Ocean (Figure 1, Table 1). Sample origins span macroalgae, lichens, sponge tissue, and sediments, reflecting the broad ecological niches that support fungal colonization in polar environments. Our sampling encompassed two distinct Antarctic environments: seven isolates were obtained from coastal sites around Robert Island within the South Shetland archipelago, while one geographically isolated sample was collected along the Graham coast of the continental Antarctic Peninsula, south of this archipelago.

Fungi taxa include *Penicillium chrysogenum* (A7) and *Penicillium crustosum* (A15A), isolated from the brown algae *Himantothallus grandifolius* (A. Gepp & E. S. Gepp) Zinova and *Desmarestia antarctica* R. L. Moe & P. C. Silva, respectively. *Cladosporium* sp. (A21F) from red alga *Gigartina skottsbergii* Setchell & N. L. Gardner and *Cladosporium malorum* (A38) from lichen *Usnea antarctica* Du Rietz. *Botrytis* sp. (A22A), isolated from green alga (seaweed) *Monostroma hariotii* Gain, and *Ulocladium microsporum* (A24B) isolated from sediments. The *Umbilicaria antarctica* Frey & Lamb and *Usnea antarctica* lichen-associated isolates *Alternaria alternata* (A29A) and *Alternaria malorum* (A36), respectively, together with the *Anoxycalyx joubini* Topsent sponge-derived *Aspergillus awamori* (A30), further expand the ecological diversity of the collection.

Several *Penicillium* species isolated from diverse marine and extreme environments have been shown to produce structurally unique and biologically active secondary metabolites, with antibacterial, antibiofilm, cytotoxic, and anti-inflammatory potential. The endophytic fungus *P. chrysogenum* AUMC14791, obtained from the soft coral *Paralemnalia* sp. from the Red Sea, yielded metabolites 15-deoxy-15-amino-citreohybridonol and chrysogenotoxin, the latter identified as a promising antibacterial agent against drug-resistant pathogens [14]. Another strain, *P. chrysogenum* VH17 from Vietnamese soft coral, produces a peptide-derived metabolite, methyl 3-((1-((2-carbamoylphenyl)amino)-1-oxopropan-2-yl)amino)-3-oxopropanoate, and an ergostane steroid, 5*α*,6*α*-epoxy-(22*E*,24*R*)-ergosta-8(14),22-diene-3*β*,7*α*-diol, the latter showing cytotoxicity toward HepG2, A549, and MCF7 cancer cells [15]. Investigations of *P. chrysogenum* WX6 from the “Caldara di Manziana” nature reserve in Italy reported pencillixanthone A, which displayed strong antibacterial activity against multiple tested strains [16]. Attia et al. [17] reported that *P. chrysogenum* extracts isolated from *Artemisia judaica* inhibited *Pseudomonas aeruginosa* biofilm formation.

Across the Antarctic continent, species such as *Penicillium* and *Aspergillus* have been shown to produce metabolites with antibacterial, antifungal, cytotoxic, anti-neuroinflammatory, and anti-allergic activities [18]. Among the most important examples are the xanthocillin derivatives, xanthocillin X and xanthocillin Y_1_, obtained from *P. chrysogenum* CCTCC M 2020019 isolated from Antarctic penguin feces, which exhibited inhibitory effects against several Gram-negative pathogens, with MIC values below 20 µg/mL [19].

Antarctic isolates of *P. crustosum* also demonstrated significant bioactivity: strain HDN153086 from Prydz Bay sediments produces a diketopiperazine with cytotoxicity against K562 cells (IC_50_ = 12.7 µM) [20], while strain PRB-2 from deep-sea sludge produces penilactones A and B, rare-skeleton metabolites with weak NF-κB inhibitory activity [21].

Mohamed and Ibrahim [22] compiled extensive data on bioactive metabolites produced by marine-derived *Cladosporium* species. In another studies, *Cladosporium* strains isolated from diverse marine regions yielded secondary metabolites with antioxidant and cytotoxic properties; however, none of the tested metabolites exhibited antibiofilm or antimicrobial activity [23,24].

In contrast, studies on *Cladosporium* sp. F14 from mangrove environments revealed a broader bioactivity profile [25] demonstrated that this strain produces metabolites with antifouling and antimicrobial activity under suitable nutrient conditions, including 3-phenyl-2-propenoic acid, cyclo-(Phe-Pro), and cyclo-(Val-Pro), which showed antibacterial effects against fouling bacteria. Additionally, 3-phenyl-2-propenoic acid and bis(2-ethylhexyl)phthalate inhibited larval settlement of *Bugula neritina* and *Balanus amphitrite*, highlighting their potential as natural antifouling agents [25].

*Botrytis* sp., isolated from the surface of the marine green alga *Enteromorpha compressa* (Linnaeus) Nees in Busan, South Korea, produced the cyclopentenone derivatives, bromomyrothenone B and botrytinone, which were tested for antimicrobial, tyrosinase-inhibitory, and radical-scavenging activities, but showed no detectable bioactivity [26].

To the best of our knowledge, no bioactive metabolites have been previously reported from *Ulocladium microsporum* strains.

*Alternaria* species from diverse environments, including saltwater lakes, mangroves, terrestrial plants, soft corals, and marine algae, have yielded a wide array of bioactive secondary metabolites with antimicrobial, antibiofilm, antifungal, antiviral, cytotoxic, and ecological activities. *Alternaria alternata* 13A from a Saharan saltwater lake produced both known metabolites and the metabolites, phragamide A and B, with four purified metabolites showing strong antibiofilm activity at 100 µg/mL; phragamides A and B, tenuazonic acid, and altechromone A inhibited Gram-positive strains by 70–80% and Gram-negative strains by 40–60% [27]. Additional work on *Alternaria* strains EL 24 and EL 35 from *Eremophila longifolia* leaves demonstrated 57–72% inhibition of biofilm formation and 54–64% reduction in pre-formed biofilms against MRSA and MSSA [28]. Extracts from *A. alternata* associated with *Carica papaya* also significantly reduced biofilm formation, likely through effects on exopolysaccharide production and cell surface hydrophobicity [29].

Marine-associated *Alternaria* strains have shown additional bioactivities: pyrophen and rubrofusarin B from *A. alternata* linked to the Red Sea soft coral *Dendronephthya hemprichi* Klunzinger exhibited antifungal activity against *Candida albicans* [30]. Alternariol derivatives from *A. alternata*, inhabiting *Litophyton arboreum,* inhibited HCV-NS3/NS4A protease, with alternariol showing activity (IC_50_ = 12.0 µg/mL) [31]. Sesteralterin and tricycloalterfurenes A–D from *A. alternata* associated with the red alga *Lomentaria hakodatensis* Yendo inhibited the growth of marine plankton [32]. Finally, alternariethers A–C and alternariester from *Alternaria malorum*, a mangrove endophyte of *Myoporum bontioides* (Siebold & Zucc.) A. Gray, displayed antifungal activity against plant pathogens *Fusarium oxysporum* and *F. graminearum* [33].

*Aspergillus awamori*, collected from the River Nile habitat in Egypt in 2015, yielded phthalate (DEHP), which exhibited cytotoxic activity against the MCF7, HepG2, HCT116, and HeLa cancer cell lines, with IC_50_ values ranging from 6.5 to 66.6 µg/mL [34].

The above mentioned studies highlight the Antarctic isolates investigated here as part of a broader global pattern: marine-derived polar fungi constitute a rich and still largely untapped reservoir of chemically diverse metabolites with promising bioactivity potential. The ecological uniqueness of Antarctic habitats, combined with the demonstrated bioactivity of related fungal taxa worldwide, reinforces the value of these isolates as compelling candidates for future natural-product discovery and biotechnological exploration.

### 2.2. Antimicrobial Activity Evaluation

Three of the nine Antarctic marine-derived fungal extracts tested, *Aspergillus awamori* (A30), *Ulocladium microsporum* (A24B), and *Cladosporium malorum* (A38), showed low antimicrobial activity against MRSA strain COL and MSSA strain NCTC8325-4 (Table 1). These antimicrobial activities were restricted to Gram-positive bacteria, with A30 and A38 showing minimum inhibitory concentrations (MIC) of 125 µg/mL for MRSA and A24B and A38 showing MICs of 125 µg/mL and 250 µg/mL for MSSA, respectively. None of the extracts inhibited the growth of *E. coli*.

Previous studies reported that *A. awamori* F12 produces antimicrobial metabolites, such as emodin, an anthraquinone derivative highly effective against various clinical and foodborne pathogens, including MRSA strains [35]. *U. microsporum* has not been previously reported as an antimicrobial producer. Similarly, most marine-derived *Cladosporium* strains examined to date showed no antimicrobial activity, with the exception of the mangrove strain *Cladosporium* sp. F14 [25].

### 2.3. Anticancer Activity Evaluation

**Figure 2:**
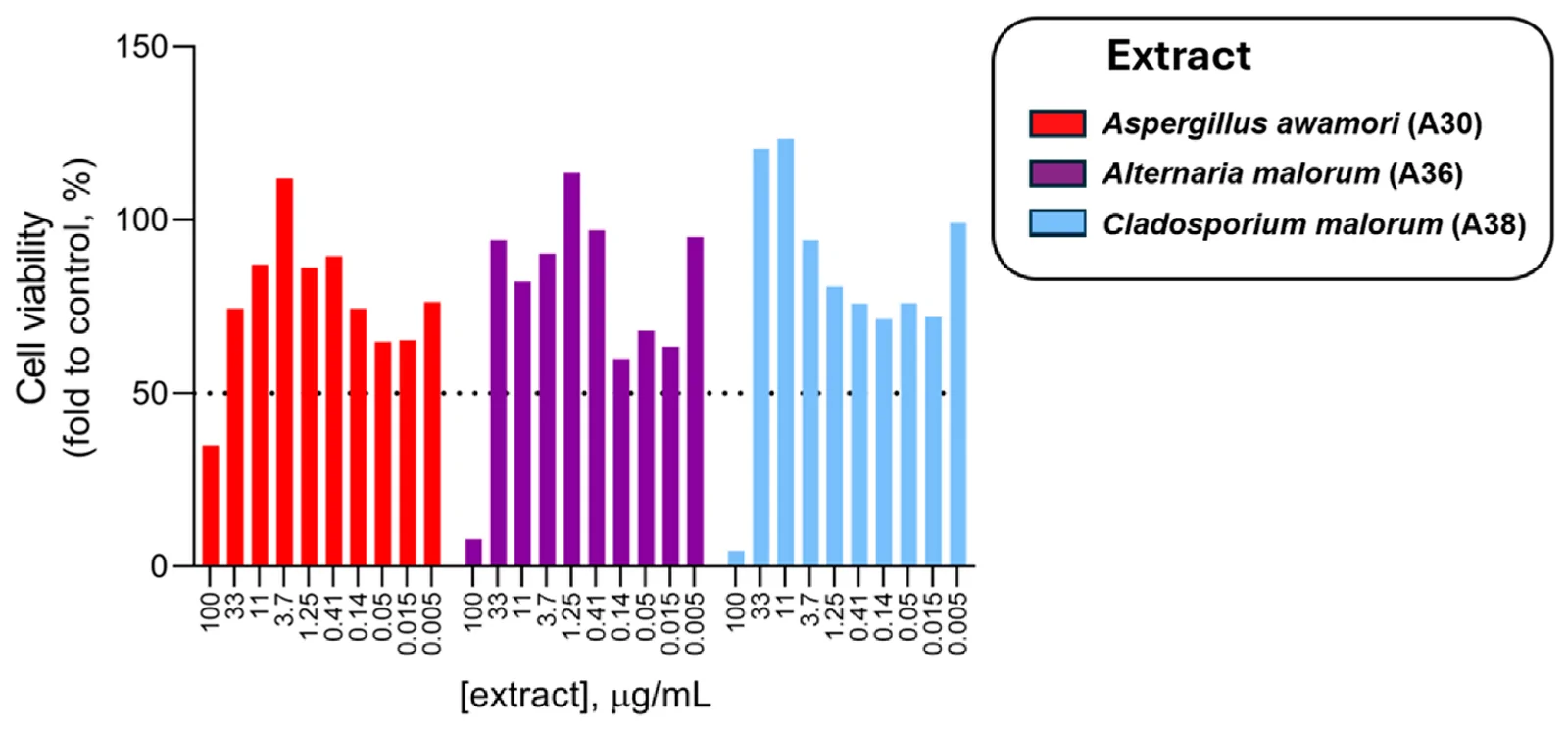
HCT-116 cell viability following 72 h incubation with Antarctic fungal extracts from *A. awamori* (A30), *A. malorum* (A36), and *C. malorum* (A38). Cell viability was assessed using the MTS metabolic assay. DMSO was used as vehicle control. *n* = 1.

Cytotoxicity toward human colorectal adenocarcinoma cells (HCT-116) was observed for *Aspergillus awamori* (A30), *Alternaria malorum* (A36), and *Cladosporium malorum* (A38), each with estimated IC_50_ values ≥30 µg/mL (Table 1, Figure 2). According to established potency thresholds, both antimicrobial and cytotoxic activities fall within the moderately active range (10–250 µg/mL), remaining above the levels typically required for lead-metabolite development (<10 µg/mL for extracts; <1 µg/mL for pure metabolites) [36].

### 2.4. Antibiofim Activity Evaluation

**Figure 3:**
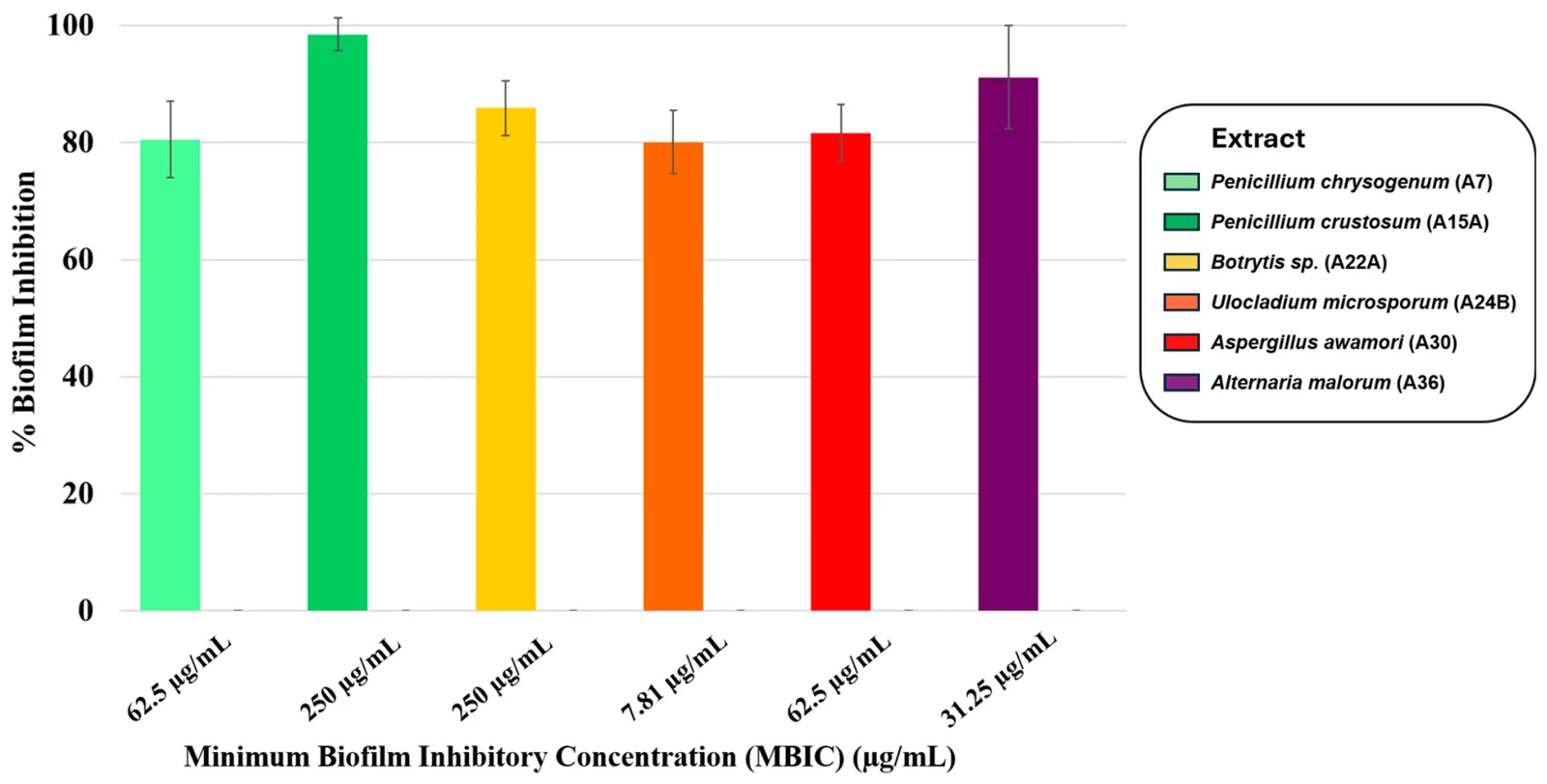
Inhibition of biofilm formation of MSSA (NCTC8325-4 upon growth with the MBIC concentration of Antarctic fungal extracts of *P. chrysogenum* (A7), *P. crustosum* (A15A), *Botrytis* sp. (A22A), *U. microsporum* (A24B), *A. awamori* (A30), and *A. malorum* (A36). Cultures grown with 2.5% DMSO (positive control) were considered to represent 100% of viability and biofilm formation. Growth media corresponds to negative control. Shown are means (*n* = 3) with the standard error of the mean (SEM).

The antibiofilm results obtained against the MSSA strain revealed that six of the nine extracts tested exhibited antibiofilm activity (Figure 3). To the best of our knowledge, no Antarctic fungi have been previously reported to produce secondary metabolites with antibiofilm activity.

The percentage of biofilm inhibition ranged from 81.10% to 98.50%, indicating that *P. chrysogenum* (A7), *P. crustosum* (A15A), *Botrytis* sp. (A22A), *U. microsporum* (A24B), *A. awamori* (A30), and *A. malorum* (A36) extracts possessed strong antibiofilm potential at several minimum biofilm inhibitory concentrations (MBIC). In addition, all these extracts did not inhibit planktonic bacterial growth, thus possessing potent antibiofilm activity against NCTC 8325-4 strain.

In detail, *P. crustosum* (A15A) exhibited the highest antibiofilm activity, achieving 98.50% biofilm inhibition for an extract concentration of 250 µg/mL. *A. malorum* (A36) demonstrated the second-highest inhibitory activity (91.16% at 31.35 µg/mL), also indicating excellent antibiofilm potential.

*Botrytis* sp. (A22A) showed 85.89% of biofilm inhibition for 250 µg/mL, further indicating potent antibiofilm efficacy. *P. chrysogenum* (A7) and *U. microsporum* (A24B) inhibited biofilm formation by 81.69% and 81.10%, at 62.5 µg/mL and 7.81 µg/mL, respectively.

These results are particularly relevant, as all the extracts inhibited over 80% of biofilm formation, while presenting a growth inhibitory activity below 40%, indicating selective interference with biofilm formation rather than general antibacterial toxicity. Metabolites that show these levels of inhibition of biofilm and planktonic growth are considered clinical antibiofilm “hits” [36,37]. This supports the potential of Antarctic fungi as promising sources of natural antibiofilm agents for combating persistent infections associated with biofilm-forming pathogens.

Antibiofilm activities associated with *P. chrysogenum* and *Alternaria* species, such as *A. alternata* have previously been described [17,27,28]. In contrast to the studies described in Section 2.1, in our study, *A. alternata* (A29A) extract showed no antibiofilm activity, suggesting a distinct metabolite profile from those previously reported. Moreover, antibiofilm activity is rare or absent in strains of the genera *Botrytis, Ulocladium*, and *Aspergillus* [23,24].

Our findings highlight the potential of Antarctic fungi as a valuable source of novel antibiofilm metabolites and reinforce the importance of exploring extremophilic microorganisms for the discovery of alternative strategies against biofilm-associated infections.

### 2.5. Molecular Network of Fungal Isolates

**Figure 4:**
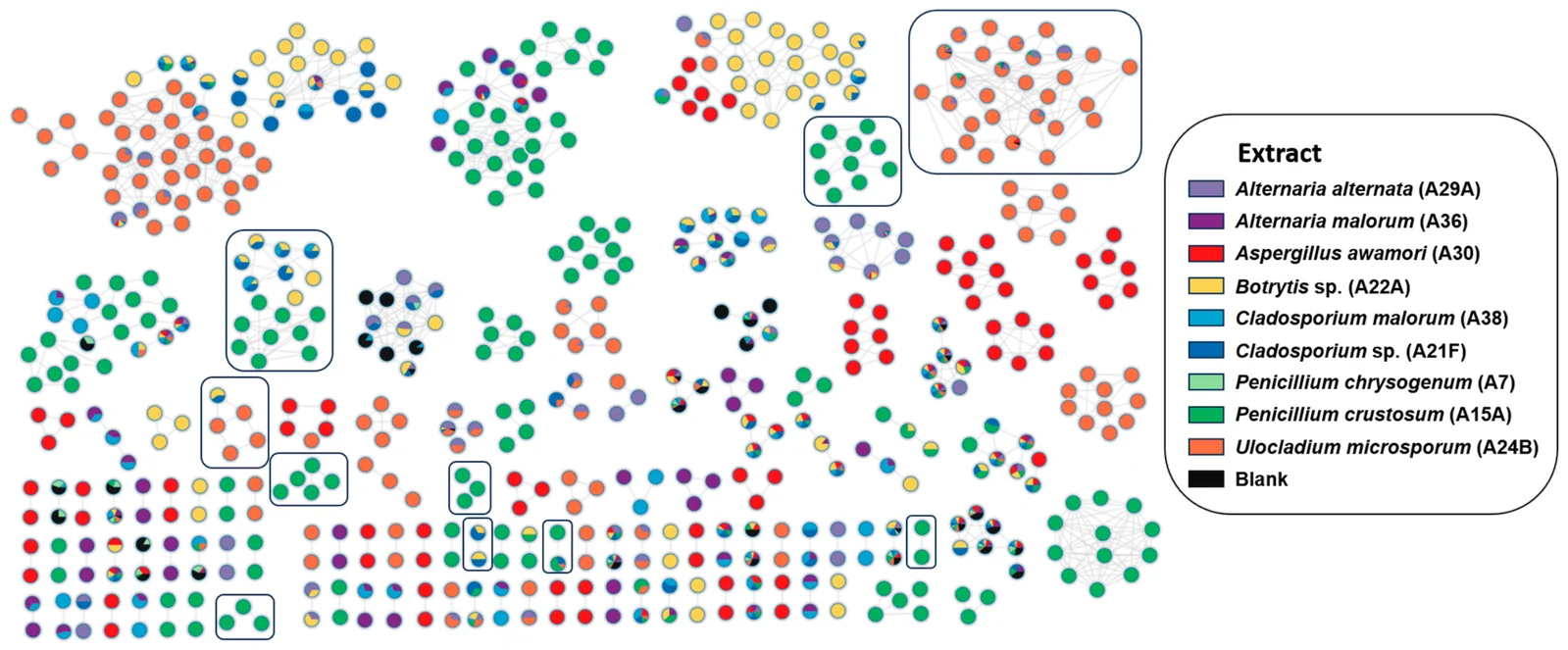
Molecular networking for Antarctica fungi species using MS/MS data (ESI+). Nodes represent parent ions, and edge indicates the chemical similarity between the MS/MS spectra. Node colors represent the species according to the legend. Only networks containing at least two nodes are shown. Nodes highlighted in black boxes will be presented in Figure 5 and Figure 6.

Using the LC–MS/MS dataset, a molecular network was generated on the GNPS platform for the nine fungal extracts (Figure 4). The resulting network contained 1360 nodes (ions), of which 80 (approximately 5.88%) were successfully annotated through spectral matches to known metabolites in the GNPS libraries, enabling efficient dereplication [38,39].

*P. crustosum* biosynthetic clusters predominate in the molecular network, followed by those of *U. microsporum* and *A. awamori*, each revealing multiple distinct molecular families as well as species-specific metabolite classes (Figure 4).

The GNPS molecular networking suggests the existence of unexplored chemical diversity within these fungi. Many clusters have no annotations, indicating that several molecular families may contain previously undescribed metabolites, representing a promising reservoir for future natural product discovery and biosynthetic investigation.

**Figure 5:**
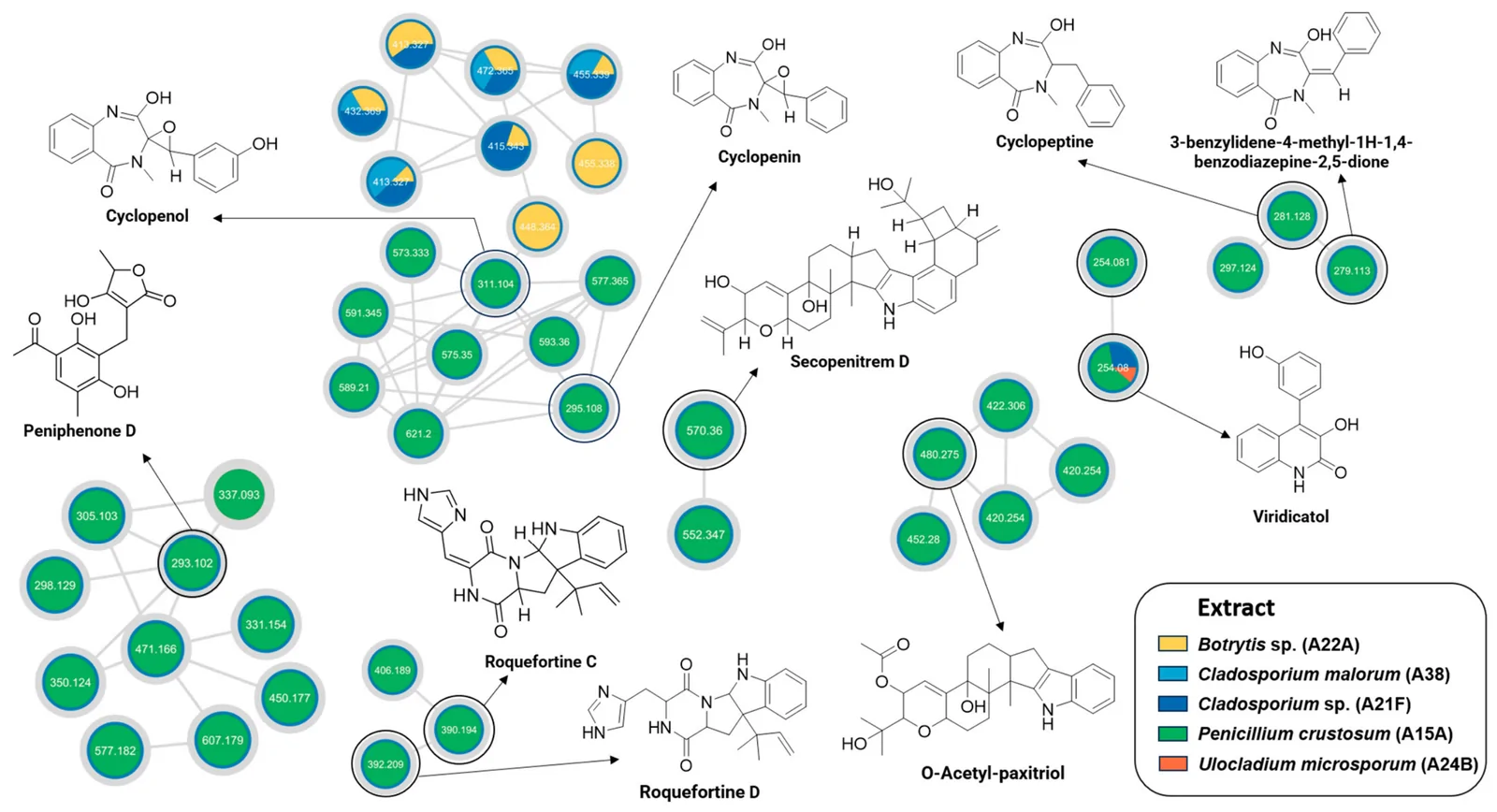
Clusters for roquefortine-, peniphenone-, cyclopenol-, cyclopeptine-, and o-acetyl-paxitriol-derivatives produced by Antarctic fungi. Node colors represent the extracts according to the legend. Nodes represent parent ions, and edge strength indicates the chemical similarity between the MS/MS spectra. Nodes highlighted in black circles presented a spectral match with the library.

Several molecular family clusters identified by the GNPS molecular networking are highlighted in Figure 5, mainly associated with *P. crustosum*. The annotated molecular families include roquefortine-, peniphenone-, cyclopenol-, cyclopeptine-, and o-acetyl-paxitriol-derivatives, demonstrating the high chemical diversity of this Antarctic fungal isolate.

The roquefortine cluster is particularly relevant because these alkaloids are well-known *Penicillium* sp. metabolites associated with antimicrobial and cytotoxic activities [40,41,42]. Similarly, cyclopenin, cyclopenol, and cyclopeptine derivatives belong to quinoline- and benzodiazepine-related alkaloids previously reported with antimicrobial, antifungal, and enzyme inhibitory properties [43,44,45,46]. The peniphenone derivatives further support the pharmaceutical potential due to their reported antimycobacterial activity [47].

The presence of o-acetyl-paxitriol and related indole diterpenoid metabolites suggests the existence of complex biosynthetic pathways capable of producing structurally sophisticated fungal natural products.

**Figure 6:**
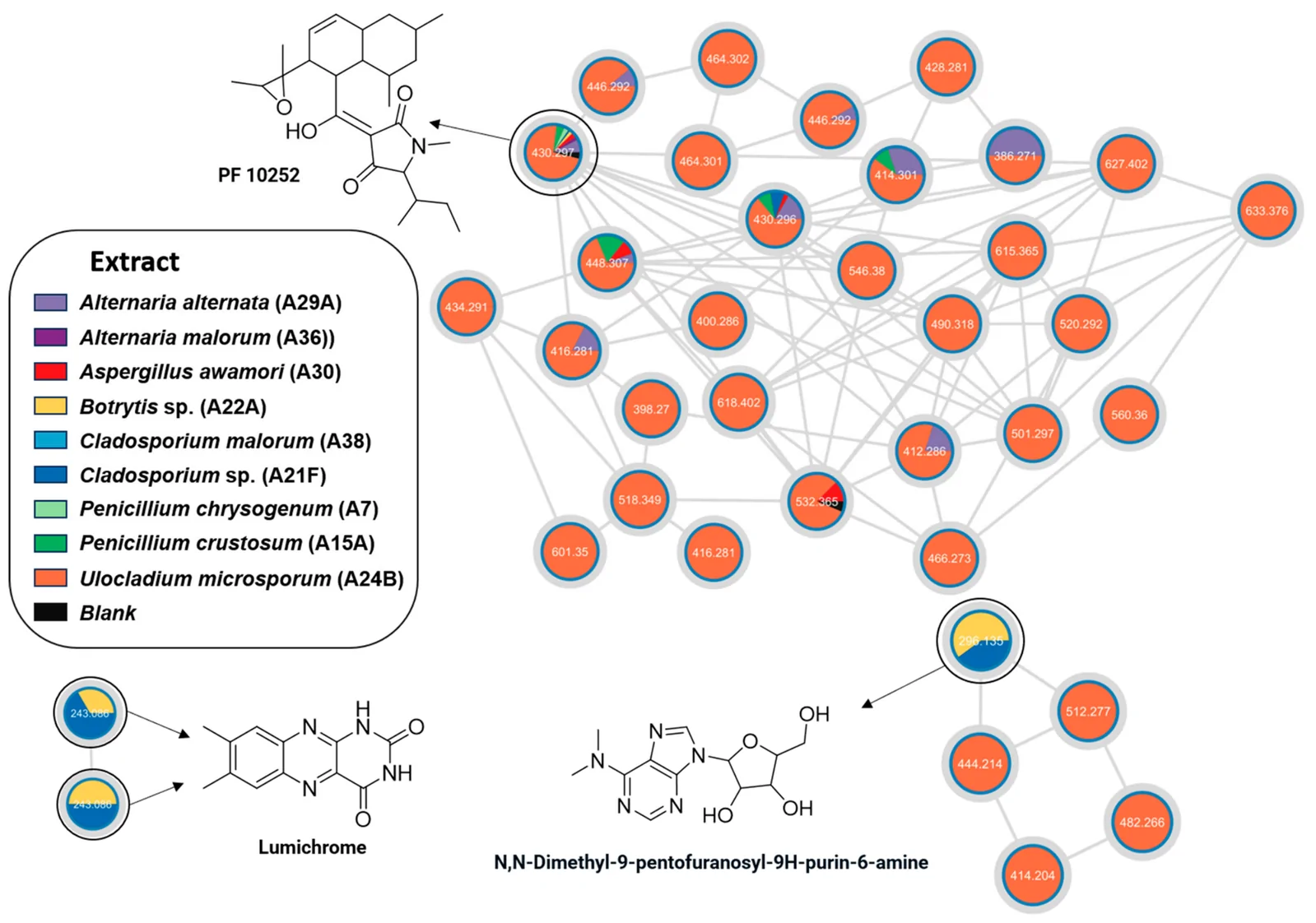
Clusters for PF 10252, lumichrome, and nucleoside derivatives produced by Antarctic fungi. Node colors represent the extracts according to the legend. Nodes represent parent ions, and edge strength indicates the chemical similarity between the MS/MS spectra. Nodes highlighted in black circles presented a spectral match with the library.

Molecular family clusters corresponding to PF1052, lumichrome, and nucleoside derivatives were annotated across several Antarctic fungal isolates, particularly *U. microsporum*, *Botrytis* sp., and *Cladosporium* sp., highlighted in Figure 6. Unlike the metabolite families in Figure 5, these metabolites were shared among phylogenetically distinct fungi, suggesting conserved metabolic adaptations to Antarctic environmental conditions.

PF1052-related metabolites are known antibiotic agents produced by fungi *Phoma* sp. [48]. The lumichrome cluster, identified in *Botrytis* sp. and *Cladosporium* sp., is associated with oxidative stress adaptation, plant-growth promotion, and anticancer activity, indicating possible ecological functions linked to survival in extreme polar environments [49].

The nucleoside derivative clusters contained modified purine-related metabolites that may be involved in signalling, metabolic regulation, or stress-response mechanisms.

These findings demonstrate that Antarctic fungi produce diverse and potentially unique metabolite families beyond classical fungal alkaloids and polyketides, reinforcing their importance as promising sources of chemically novel natural products with ecological and pharmaceutical relevance.

### 2.6. Metabolomic Profiling of Fungal Isolates

Untargeted LC–MS/MS metabolomic profiling revealed a highly diverse repertoire of secondary metabolites across the investigated fungal taxa, spanning six major chemical classes: alkaloids, polyketides, terpenoids/meroterpenoids, peptides (including diketopiperazines), nucleosides, and a heterogeneous group of mixed metabolite classes (Table 2).

Among the analyzed fungi, *P. crustosum* exhibited the highest chemical diversity, producing metabolites encompassing nearly all detected chemical classes, including alkaloids, polyketides, meroterpenoids, indole diterpenoids, diketopiperazines, and carotenoid-related metabolites, indicating an extensive and metabolically versatile secondary metabolite profile. The simultaneous detection of roquefortines, viridicatin-related alkaloids, pyripyropene derivatives, andrastins, secopenitrems, and diketopiperazines within a single strain is particularly relevant, as these metabolite families originate from distinct biosynthetic pathways and are not commonly reported together in the same isolate. Such chemical diversity suggests the presence of multiple active biosynthetic gene clusters and highlights this strain as a promising source of bioactive natural products.

As mentioned above, another distinctive feature of the dataset was the occurrence of several metabolites across phylogenetically unrelated fungal genera, including *Botrytis* sp., *Cladosporium* sp., *A. awamori*, and *U. microsporum*. Metabolites such as cyclopenin, viridicatol, polanrazine B, lumichrome, and carotenoid derivatives were repeatedly detected among multiple isolates. In particular, the identification of polanrazine B across several genera is unusual and may indicate a broader but underreported occurrence of this metabolite family in fungal systems.

Alkaloids represented the most abundant annotated class, including roquefortines, cyclopenins, cyclopeptines, viridicatins, and related quinoline/benzodiazepine derivatives. Several of these metabolites, such as meleagrin, roquefortine C, cyclopenin, and viridicatin, have previously been reported from *Penicillium* species and are associated with antimicrobial, cytotoxic, antifungal, or enzyme inhibitory activities (Table 2).

Roquefortine alkaloids are widely recognized as chemotaxonomic markers in *Penicillium* species and have been associated with defensive ecological functions against microbial competitors [42]. Similarly, cyclopenin and viridicatol derivatives are known fungal quinoline alkaloids with documented acetylcholinesterase inhibitory, antifungal, and antiallergic activities, highlighting the pharmaceutical relevance of these isolates [57,58].

Polyketide metabolites such as altersolanol A, peniphenone D, and fonsecin were detected in several isolates. Altersolanol A, annotated from *A. malorum*, has previously demonstrated cytotoxic and antibacterial activities, supporting the therapeutic potential of metabolites produced by *Alternaria* species [61,62]. Peniphenone D, associated with antimycobacterial activity, further emphasizes the capacity of fungal-derived polyketides to yield medically relevant scaffolds [47].

Terpenoid and meroterpenoid annotations included andrastin A, pyripyropene A derivatives, secopenitrem D, and polanrazine B. These metabolites were predominantly associated with *P. crustosum*, though cross-genus occurrence was also observed in *A. awamori*, *Cladosporium* sp., and *Botrytis* sp. Meroterpenoids are biosynthetically significant because they arise from hybrid terpene–polyketide pathways that are commonly associated with structurally complex fungal metabolites. The identification of andrastin A is particularly relevant due to its reported anticancer activity and its classification as a meroterpenoid with intricate biosynthetic origins [65].

Cyclic dipeptides and related peptide-derived metabolites, including histidyltryptophyl diketopiperazine, fusaperazine E, pyroGlu-Phe, and additional indole-containing piperazine-2,5-dione derivatives, were annotated in *P. crustosum* and *A. awamori*. PyroGlu-Phe was reported for anti-inflammatory activity, suggesting potential biomedical applications for simple fungal peptides that are often overlooked in natural product studies [70].

Nucleoside-like metabolites were primarily associated with *U. microsporum*, *Botrytis* sp., and *Cladosporium* sp., including purine nucleosides derivatives containing hydroxymethyl and dimethylamino substitutions.

The “other/mixed” category contained structurally diverse metabolites lumichrome, formononetin, carotenoid derivatives, and a putative ofloxacin-like structure. The detection of plant-associated flavonoids (e.g., formononetin) and synthetic antibiotic-like metabolites (e.g., ofloxacin) likely reflects either environmental uptake, co-cultured microbial communities, or spectral misannotation within GNPS database.

Our findings indicate that the investigated fungal strains, particularly *P. crustosum*, represent promising sources of chemically diverse and potentially novel secondary metabolites with ecological and pharmaceutical significance.

### 2.7. Correlation of Annotated Metabolites with Bioactivity

Metabolites previously reported to possess cytotoxic or anticancer properties, including altersolanol A, alkaloids such as meleagrin, and meroterpenoid derivatives were annotated in the species that exhibited cytotoxic activity against HCT-116 cells (Section 2.3). Similarly, the presence of metabolites such as roquefortine C, viridicatin, and viridicatol, previously associated with antimicrobial effects, were detected in strains that showed antimicrobial activity against MRSA and MSSA (Section 2.2). Although biological activities observed in this study were partially consistent with the annotated metabolite profiles obtained by GNPS analysis, the absence of bioactivity-guided fractionation and metabolite isolation prevents direct attribution of the observed effects to specific annotated metabolites.

No antibiofilm studies have previously been reported for the annotated metabolites identified in these strains. Therefore, while the metabolomic analysis confirms the presence of chemically and biologically relevant secondary metabolites, the metabolites potentially responsible for the antibiofilm activity remain unknown. Consequently, these strains represent promising candidates for future bioactivity-guided isolation studies targeting novel antibiofilm metabolites.

## 3. Material and Methods

### 3.1. Isolation of Marine-Derived Fungi

Different marine organisms were collected from the oceans and regional seas surrounding Antarctica in April 2018, (Table 1). Scuba diving was employed to collect marine organisms, which were then transferred to containers. To minimize bacterial growth in the culture medium, fungal isolation was processed immediately. Following the protocol established by Kjer et al. [80], samples were cut into 1 × 1 cm pieces and subjected to a triple rinse with sterile water to remove surface debris. Subsequently, the samples were sterilized by immersion in 70% ethanol (*v*/*v*) for 60–120 s. Following drying, the samples were transferred to culture medium containing SDA and artificial sea salt. These cultures were incubated for 5–7 days at 25 °C under light conditions. Fungal isolates were stored at −80 °C for other studies as 3 replicates [81]. A total of nine Antarctic fungal isolates were obtained from distinct ecological substrates: four isolates from macroalgae, three from lichens, one from sponge tissue, and one from a sediment sample.

### 3.2. Identification of Fungi

Fungal strain identification was achieved through DNA isolation utilizing the Biospeedy^®^ Fungal Diversity Kit (Bioeksen, Istanbul, Turkey), a fungal DNA isolation and PCR-based system. A pair of forward primers (TCCTCCGCTTATTGATATGC) and reverse primers (GGAAGTAAAAGTCGTAACAAGG) specific to the ITS1-5.8S-ITS2 region were used [82]. The sequences of the amplified fungal DNA were determined using the ABI Prism BigDye Terminator Cycle Sequencing Ready Reaction Kit on an ABI Prism 377 DNA Sequencer [81,83].

### 3.3. Preparation of Fungal Extracts

For large-scale production of fungal extracts, 2 L Erlenmeyer flasks containing sterilized rice medium and artificial sea salt medium were inoculated with fungal strains. The cultures were incubated for 4–6 weeks at 25 °C. Fungal extraction was performed using ethyl acetate (EtOAc) via maceration repeated three times. Total extract was evaporated under vacuum and kept at 4 °C for further use [80]. A 10 mg/mL solution in DMSO for each crude extract was performed for subsequent bioassay testing.

### 3.4. Antimicrobial Assays

The broth microdilution method previously reported by group was used to evaluate the antimicrobial activity of the nine fungal extracts [84,85]. Minimum inhibitory concentrations (MICs) against methicillin-resistant *S. aureus* COL (MRSA), methicillin-susceptible *S. aureus* NCTC8325-4 (MSSA), and *E. coli* K12 were determined. The bacterial strains were cultured in Mueller–Hinton broth medium (MHB) (Difco Laboratories, Detroit, MI, USA).

Each extract was solubilized in DMSO to a concentration of 10 mg/mL and sequentially diluted 2-fold in fresh bacterial culture with an initial OD620nm of 0.05, in flat-bottom polystyrene 96-well microplates (Labbox, Barcelona, Spain), to a range of final concentrations of 250 to 7.81 µg/mL. The microplate was incubated for 18 h at 37 °C without agitation. The positive control consisted of bacterial culture supplemented with DMSO. The assays were performed in triplicate.

### 3.5. Antibiofilm Assays

Static biofilm assays were performed as previously described by our group [8]. *S. aureus* NCTC 8325-4 was inoculated into fresh TSB supplemented with 1% glucose, to an initial OD620nm of 0.05. The same fungal extracts used for the antimicrobial assays were sequentially diluted 2-fold in the NCTC 8325-4 culture, in flat-bottom Nunclon delta-treated 96 well polystyrene microplate (ThermoFisher Scientific, Waltham, MA, USA), to a range of final concentrations of 250 to 7.81 µg/mL. The microplate was incubated at 37 °C for 18 h without agitation and the optical density was measured using a SpectraMax 190 Microplate Reader (Molecular Devices, New York, NY, USA). The 96-well microplate was washed thrice with double distilled water to remove the planktonic cells, and the biofilm was fixed by incubation at 60 °C for 1 h. A crystal violet solution (0.06% *w*/*v*) was added to the wells to stain the biofilm, and after three washes with double distilled water, the biofilm was resuspended in 30% acetic acid and quantified by measuring the OD620nm. As a positive control DMSO was added instead of extract. Biofilm inhibition percentages for the different concentrations of the extracts were retrieved relatively to the biofilm produced in the positive control. The assays were performed in triplicate, and the results represent the mean and the standard error of the mean.

### 3.6. Anticancer Assays

HCT-116 human colorectal adenocarcinoma cells were plated in 96-well plates (5000 cells/well) for 24 h and then incubated with marine extracts (doses ranging from 100 to 0.0051 μg/mL), vehicle control (DMSO, 1%) or positive control (5-FU, 10 μM), according with previously described methodology by our group [84]. After 72 h of incubation, cell viability was assessed based on the measurement of 3-(4,5-dimethylathiazol-2-yl)-5-(3-carboxymethoxyphenyl)-2-(4-sulfophenyl)-2H-tetrazolium, inner salt (MTS) metabolism using CellTiter 96^®^ Aqueous Non-Radioactive Cell Proliferation Assay (Promega, Madison, WI, USA). IC_50_ best fit values were determined using the log-(inhibitor) vs. response-variable slope (four parameters) function, using GraphPad Prism 9 software (La Jolla, San Diego, CA, USA). The assays were performed in single measurements at each concentration.

### 3.7. LC–MS/MS Analysis

Actinomycete crude extracts were diluted in methanol (1.0 mg/mL) and analyzed by LC–MS/MS. The analyses were carried out on an ELUTE + UHPLC (Bruker, Berlin, Germany) coupled to an Impact II QqTOF mass spectrometer with electrospray ionization (ESI+), using positive ionization mode. Chromatographic separation was performed on an Kinetex ^®^ column (1.7 μm C18, 100 × 2.1 mm) at 40 °C. Water and acetonitrile were used as mobile phases A and B, respectively, both containing 0.1% formic acid. The chromatographic separation was performed using a gradient elution at a flow rate of 0.5 mL min^−1^. The mobile phase composition started at 10% solvent B and was linearly increased to 60% B over 3 min. From 3 to 8 min, solvent B was further increased to 100%, followed by a 1 min hold at 100% B. The composition was then returned to 10% B between 9.0 and 9.1 min and maintained under these initial conditions until the end of the run at 10 min, with an injection volume of 3 µL. Ion source parameters were: endplate: 500 V, capillary voltage: 4500 V, drying temperature: 250 °C, drying gas: 9.0 L/min, *m*/*z* range: 100–1500, gas pressure: 40 psi. An untargeted method was used in the spectrometer in which ions of higher intensity were selected for fragmentation. A ramp from 20 to 70 eV was used for collision energy.

### 3.8. Molecular Networking

A molecular network was created using the online workflow (https://wang-bioinformatics-lab.github.io/GNPS2_Documentation/; accessed on 15 May 2026) on the GNPS2 website (https://gnps2.org/homepage) [86]. The data was filtered by removing all MS/MS fragment ions within +/− 17 Da of the precursor *m*/*z*. MS/MS spectra were window filtered by choosing only the top 6 fragment ions in the +/− 50 Da window throughout the spectrum. The precursor ion mass tolerance was set to 0.02 Da and a MS/MS fragment ion tolerance of 0.02 Da. A network was then created where edges were filtered to have a cosine score above 0.65 and more than 4 matched peaks. Further, edges between two nodes were kept in the network if and only if each of the nodes appeared in each other’s respective top 10 most similar nodes. Finally, the maximum size of a molecular family was set to 100, and the lowest scoring edges were removed from molecular families until the molecular family size was below this threshold. The spectra in the network were then searched against GNPS2’ spectral libraries. The library spectra were filtered in the same manner as the input data. All matches kept between network spectra and library spectra were required to have a score above 0.65 and at least 4 matched peaks.

## 4. Conclusions

This study demonstrates that Antarctic marine-derived fungi constitute a chemically rich and biologically relevant source of natural products with antibiofilm and anticancer potential.

The most significant outcome of this work was the strong and highly selective antibiofilm activity exhibited by six of the nine fungal isolates. All extracts inhibited biofilm formation by 81.1–98.5% while not affecting planktonic bacterial growth, indicating antibiofilm “hits” under clinical development criteria. Among them, *P. crustosum* (A15A) exhibited the highest level of inhibition.

Furthermore, three extracts, *A. awamori* (A30), *A. malorum* (A36), and *C. malorum* (A38), also exhibited potential cytotoxicity toward HCT-116 colorectal cancer cells.

Untargeted LC–MS/MS molecular networking revealed a diverse metabolome dominated by alkaloids, but also including polyketides, terpenoids/meroterpenoids, peptides (including diketopiperazines), nucleosides, and metabolites from other classes. *P. crustosum* exhibited particularly high biosynthetic plasticity, functioning as a central metabolic source producing representatives of nearly all annotated chemical classes. In contrast, strains from genera *Cladosporium*, *Botrytis*, and *Alternaria* displayed more restricted but overlapping metabolite profiles, suggesting ecological convergence in metabolite function rather than strict taxonomic specificity.

Overall, these findings highlight the value of Antarctic fungi as promising reservoirs of bioactive metabolites and reinforce the importance of continued exploration of polar microbiomes for the discovery of antibiofilm agents, expanding the current understanding of polar fungal diversity.

## Figures and Tables

**Table 1:** Antimicrobial and anticancer activity results for the Antarctic fungal extracts. NA—No activity.

Extract Code	Species	Marine Antarctic Source	Bioresource Collection Location	MRSA COL MIC µg/mL	MSSA NCTC8325-4 MIC µg/mL	*E. coli* K12 MIC µg/mL	HCT-116 IC_50_ µg/mL
			Latitude, Longitude				
A7	*Penicillium chrysogenum*	Macroalgae *Himantothallus grandifolius*	62°22′33.02″ S, 59°41′11.21″ W	NA	NA	NA	NA
A15A	*Penicillium crustosum*	Macroalgae *Desmarestia* *antarctica*	62°22′31.50″ S, 59°38′19.73″ W	NA	NA	NA	NA
A21F	*Cladosporium* sp.	Macroalgae *Gigartina* *skottsbergii*	62°21′12.74″ S, 59°39′37.07″ W	NA	NA	NA	NA
A22A	*Botrytis* sp.	Macroalgae (seaweed) *Monostroma* *hariotii*	62°21′12.74″ S, 59°39′37.07″ W	NA	NA	NA	NA
A24B	*Ulocladium* *microsporum*	Sediments	62°20′37.53″ S, 59°37′15.73″ W	NA	125	NA	NA
A29A	*Alternaria* *alternata*	Lichen *Umbilicaria* *antarctica*	62°21′29.56″ S, 59°40′27.42″ W	NA	NA	NA	NA
A30	*Aspergillus awamori*	Sponge *Anoxycalyx joubini*	64°36′53.11″ S, 62° 5′7.97″ W	125	NA	NA	≥30
A36	*Alternaria* *malorum*	Lichen *Usnea antarctica*	62°22′53.31″ S, 59°39′42.09″ W	NA	NA	NA	≥30
A38	*Cladosporium malorum*	Lichen *Usnea antarctica*	62°23′25.18″ S, 59°39′56.42″ W	125	250	NA	≥30

**Table 2:** Distribution of fungal secondary metabolites across chemical classes, producing species, and reported bioactivities and other fungi sources.

Chemical Class	Fungi Species	GNPS Annotated Metabolite	Previously Reported Fungi Sources	Bioactivity
Alkaloids	*Penicillium* *chrysogenum* *Botrytis* sp.	Meleagrin	*Penicillium* sp. [50]	Cytotoxic activity [51]
	*Penicillium crustosum*	Roquefortine C	*Penicillium* sp. [41]	Antimicrobial and cytotoxic activity [42]
	*Penicillium crustosum*	Roquefortine D	*Penicillium* sp. [40]	-
	*Penicillium crustosum* *Botrytis* sp. *Cladosporium malorum* *Cladosporium* sp.	Cyclopenin	*Penicillium cyclopium* [43]	AChE inhibitory activity [46]
	*Penicillium crustosum*	Cyclopeptine	*Penicillium cyclopium* [43,52]	-
	*Penicillium crustosum*	Cyclopenol	*Penicillium* sp. [44]	Antimicrobial and cytotoxic activity [45]
	*Penicillium crustosum* *Botrytis* sp. *Cladosporium malorum* *Cladosporium* sp.	Benzodiazepine derivative	-	-
	*Penicillium crustosum* *Cladosporium* sp.	Viridicatin	*Penicillium viridicatum* [53]	Antimicrobial activity [54]
	*Penicillium crustosum*	Dehydrocyclopeptine	*Penicillium* sp. [55]	-
	*Penicillium crustosum* *Cladosporium* sp. *Ulocladium* *microsporum*	Viridicatol	*Penicillium griseofulvum* [56]	Antifungal and antiallergic activity [57,58]
	*Ulocladium* *microsporum*	PF 1052	*Phoma* sp. [48]	Antibiotic activity [48]
	*Penicillium crustosum*	Quinolinone derivative	*Penicillium* sp. *Aspergillus* sp. [59]	-
Polyketides	*Alternaria malorum*	Altersolanol A	*Alternaria* sp. [60]	Cytotoxic, antibiotic activity [61,62]
	*Penicillium crustosum*	Peniphenone D	*Penicillium dipodomyicola* [47]	Antimicrobial (*Mycobacterium tuberculosis*) activity [47]
	*Aspergillus awamori*	Fonsecin	*Aspergillus* sp. [63]	Radical scavenging activity [64]
Terpenoids/Meroterpenoids	*Penicillium crustosum*	Andrastin A	*Penicillium* sp. [65]	Anticancer activity [66]
	*Penicillium crustosum* *Aspergillus awamori* *Cladosporium* sp. *Botrystis* sp.	Polanrazine B	*Phoma* sp. [67]	-
	*Penicillium crustosum Aspergillus awamori* Multiple other	Pyripyropene A derivative	-	-
	*Penicillium crustosum*	O-acetyl-paxitriol	-	-
	*Penicillium crustosum*	Secopenitrem D	*Penicillium crustosum* [68]	-
Peptides/DKPs	*Penicillium crustosum*	Diketopiperazine derivative	-	-
	*Penicillium crustosum*	Histidyltryptophyl diketopiperazine	-	-
	*Penicillium crustosum*	Fusaperazine E	*Penicillium crustosum* [69]	-
	*Bostrustis* sp. *Aspergillus awamori*	PyroGlu-Phe	-	Anti-inflammatory activity [70]
Nucleosides	*Ulocladium* *microsporum* *Botrytis* sp. *Cladosporium* sp.	N,N-Dimethyl-9-pentofuranosyl-9H-purin-6-amine (Nucleoside derivative)	-	-
	*Botrytis* sp. *Cladosporium* sp.	2-(6-(dimethylamino)-9H-purin-9-yl)-5-(hydroxymethyl)tetrahydrofuran-3,4-diol (Nucleoside derivative)	-	-
Other/Mixed	*Cladosporium* sp.	Ofloxacin	-	Antibiotic [71]
	*Alternaria malorum*	Formononetin	-	Anticancer, neuroprotective activity [72,73]
	*Botrytis* sp.	N-acetyl-tyrosine	-	Mitohormesis trigger activity [74,75,76]
	*Multiple* sp.	AC1L1X1Z Neomycin-like aminoglycoside derivative	-	-
	*Cladosporium* sp. *Botrytis* sp.	Lumichrome	*Aspergillus fumigatus* [77]	Plant growth promotor [49] Anticancer [78,79]
	*Penicillium crustosum* *Cladosporium* sp. Multiple other	Carotenoid derivative	-	-

## Data Availability

The data that supports the findings of this study are available on request and at the NCBI platform under accession numbers PZ394474, PZ394478, PZ394479, PZ394484, PZ394486, PZ394492, PZ394494, PZ394495, PZ394496 available at NCBI GenBank. All LC–MS/MS data from this study are deposited in the MassIVE repository under accession MSV000102538 and accessible via DOI https://doi.org/10.25345/C5707X339, with the GNPS2 molecular networking available under Task ID 18598ff62b20463791f2c42a97ed3f48.

## References

[1] Ordonez-Enireb E., Cucalon R.V., Cardenas D., Ordonez N., Coello S., Elizalde P., Cardenas W.B. (2022). Antarctic fungi with antibiotic potential isolated from Fort William Point, Antarctica. Sci. Rep..

[2] Deng Y., Liu Y., Li J., Wang X., He S., Yan X., Shi Y., Zhang W., Ding L. (2022). Marine natural products and their synthetic analogs as promising antibiofilm agents for antibiotics discovery and development. Eur. J. Med. Chem..

[3] Tuomanen E., Durack D.T., Tomasz A. (1986). Antibiotic tolerance among clinical isolates of bacteria. Antimicrob. Agents Chemother..

[4] Worthington R.J., Richards J.J., Melander C. (2012). Small molecule control of bacterial biofilms. Org. Biomol. Chem..

[5] Jeong G.-J., Khan F., Tabassum N., Cho K.-J., Kim Y.-M. (2024). Marine-derived bioactive materials as antibiofilm and antivirulence agents. Trends Biotechnol..

[6] Lahiri D., Nag M., Dey A., Sarkar T., Pati S., Nirmal N.P., Ray R.R., Upadhye V.J., Pandit S., Moovendhan M. (2023). Marine bioactive compounds as antibiofilm agent: A metabolomic approach. Arch. Microbiol..

[7] Melander R.J., Basak A.K., Melander C. (2020). Natural products as inspiration for the development of bacterial antibiofilm agents. Nat. Prod. Rep..

[8] Bauermeister A., Pereira F., Grilo I.R., Godinho C.C., Paulino M., Almeida V., Gobbo-Neto L., Prieto-Davo A., Sobral R.G., Lopes N.P. (2019). Intra-clade metabolomic profiling of MAR4 *Streptomyces* from the Macaronesia Atlantic region reveals a source of anti-biofilm metabolites. Environ. Microbiol..

[9] Pereira F., Almeida J.R., Paulino M., Grilo I.R., Macedo H., Cunha I., Sobral R.G., Vasconcelos V., Gaudêncio S.P. (2020). Antifouling napyradiomycins from marine-derived actinomycetes *Streptomyces aculeolatus*. Mar. Drugs.

[10] Wissner J.L., Almeida J.R., Grilo I.R., Oliveira J.F., Brízida C., Escobedo-Hinojosa W., Pissaridou P., Vasquez M.I., Cunha I., Sobral R.G. (2024). Novel metabolite madeirone and neomarinone extracted from *Streptomyces aculeoletus* as marine antibiofilm and antifouling agents. Front. Chem..

[11] Casillo A., Papa R., Ricciardelli A., Sannino F., Ziaco M., Tilotta M., Selan L., Marino G., Corsaro M.M., Tutino M.L. (2017). Anti-biofilm activity of a long-chain fatty aldehyde from Antarctic *Pseudoalteromonas haloplanktis* TAC125 against *Staphylococcus epidermidis* biofilm. Front. Cell. Infect. Microbiol..

[12] von Salm J.L., Witowski C.G., Fleeman R.M., McClintock J.B., Amsler C.D., Shaw L.N., Baker B.J. (2016). Darwinolide, a new diterpene scaffold that inhibits methicillin-resistant *Staphylococcus aureus* biofilm from the Antarctic sponge *Dendrilla membranosa*. Org. Lett..

[13] Almutairi F.A., Edrada-Ebel R.A. (2025). Marine Fungal Metabolites: A promising source for antibiofilm compounds. Molecules.

[14] Mira A., Abdel Bar F.M., Foudah A.I., Aboutaleb M.H., Ibrahim T.S., Hassan A.H.E., Khalil A.T. (2025). Bio-guided discovery of antibacterial metabolites from *Penicillium chrysogenum*. J. Enzym. Inhib. Med. Chem..

[15] Anh N.M., Minh L.T.H., Linh N.T., Dao P.T., Quynh D.T., Huong D.T.M., Van Cuong P., Huyen V.T.T., Dat T.T.H. (2024). Secondary metabolites from marine fungus *Penicillium chrysogenum* VH17 and their antimicrobial and cytotoxic potential. Biosci. Biotechnol. Biochem..

[16] Lu Y., Villegas-Moreno J., Clark B.R. (2024). Secondary metabolites from *Penicillium chrysogenum* WX6 and their chemotaxonomic significance. Biochem. Syst. Ecol..

[17] Attia E.Z., Khalifa B.A., Shaban G.M., Abdelraheem W.M., Mustafa M., Abdelmohsen U.R., El-Katatny M.M.H. (2022). Discovering the chemical profile, antimicrobial and antibiofilm potentials of the endophytic fungus *Penicillium chrysogenum* isolated from *Artemisia judaica* L. assisted with docking studies. S. Afr. J. Bot..

[18] Gonçalves V.N., Carvalho C.R., Martins L.B.M., Barreto D.L.C., da Silva B.F., Queiroz S.C.N., Tamang P., Bajsa-Hirschel J., Cantrell C.L., Duke S.O., Deshmukh S.K., Takahashi J.A., Saxena S. (2024). Bioactive metabolites produced by fungi present in Antarctic, Arctic, and Alpine ecosystems. Fungi Bioactive Metabolites: Integration of Pharmaceutical Applications.

[19] Khan I., Zhang H., Liu W., Zhang L., Peng F., Chen Y., Zhang Q., Zhang G., Zhang W., Zhang C. (2020). Identification and bioactivity evaluation of secondary metabolites from Antarctic-derived *Penicillium chrysogenum* CCTCC M 2020019. RSC Adv..

[20] Liu C.-C., Zhang Z.-Z., Feng Y.-Y., Gu Q.-Q., Li D.-H., Zhu T.-J. (2019). Secondary metabolites from Antarctic marine-derived fungus *Penicillium crustosum* HDN153086. Nat. Prod. Res..

[21] Wu G., Ma H., Zhu T., Li J., Gu Q., Li D. (2012). Penilactones A and B, two novel polyketides from Antarctic deep-sea derived fungus *Penicillium crustosum* PRB-2. Tetrahedron.

[22] Mohamed G.A., Ibrahim S.R.M. (2021). Untapped potential of marine-associated *Cladosporium* species: An overview on secondary metabolites, biotechnological relevance, and biological activities. Mar. Drugs.

[23] Amin M., Zhang X.-Y., Xu X.-Y., Qi S.-H. (2020). New citrinin derivatives from the deep-sea-derived fungus *Cladosporium* sp. SCSIO z015. Nat. Prod. Res..

[24] Huang Z.-H., Nong X.-H., Liang X., Qi S.-H. (2018). New tetramic acid derivatives from the deep-sea-derived fungus *Cladosporium* sp. SCSIO z0025. Tetrahedron.

[25] Qi S.-H., Xu Y., Xiong H.-R., Qian P.-Y., Zhang S. (2009). Antifouling and antibacterial compounds from a marine fungus *Cladosporium* sp. F14. World J. Microbiol. Biotechnol..

[26] Li X., Zhang D., Lee U., Li X., Cheng J., Zhu W., Jung J.H., Choi H.D., Son B.W. (2007). Bromomyrothenone B and botrytinone, cyclopentenone derivatives from a marine isolate of the fungus *Botrytis*. J. Nat. Prod..

[27] Qader M.M., Hamed A.A., Soldatou S., Abdelraof M., Elawady M.E., Hassane A.S.I., Belbahri L., Ebel R., Rateb M.E. (2021). Antimicrobial and antibiofilm activities of the fungal metabolites isolated from the marine *endophytes Epicoccum nigrum* M13 and *Alternaria alternata* 13A. Mar. Drugs.

[28] Caruso D.J., Palombo E.A., Moulton S.E., Duggan P.J., Zaferanloo B. (2023). Antibacterial and antibiofilm activity of endophytic *Alternaria* sp. isolated from *Eremophila longifolia*. Antibiotics.

[29] Rashmi M., Meena H., Meena C., Kushveer J.S., Busi S., Murali A., Sarma V.V. (2018). Anti-quorum sensing and antibiofilm potential of *Alternaria alternata*, a foliar endophyte of *Carica papaya*, evidenced by QS assays and in-silico analysis. Fungal Biol..

[30] Shaaban M., Shaaban K.A., Abdel-Aziz M.S. (2012). Seven naphtho-γ-pyrones from the marine-derived fungus *Alternaria alternata*: Structure elucidation and biological properties. Org. Med. Chem. Lett..

[31] Hawas U.W., El-Desouky S., Abou El-Kassem L., Elkhateeb W. (2015). Alternariol derivatives from *Alternaria alternata*, an endophytic fungus residing in red sea soft coral, inhibit HCV NS3/4A protease. Appl. Biochem. Microbiol..

[32] Shi Z.-Z., Miao F.-P., Fang S.-T., Liu X.-H., Yin X.-L., Ji N.-Y. (2017). Sesteralterin and Tricycloalterfurenes A–D: Terpenes with Rarely Occurring Frameworks from the Marine-Alga-Epiphytic Fungus Alternaria alternata *k21-1*. J. Nat. Prod..

[33] Fan W., Tao Y., Jiang C., Huang Q., Li J., Ding W., Li C. (2024). Four New Isocoumarins from the mangrove fungus *Alternaria malorum* with antimicrobial activities. Chem. Biodivers..

[34] Lotfy M.M., Hassan H.M., Hetta M.H., El-Gendy A.O., Mohammed R. (2018). Di-(2-ethylhexyl) Phthalate, a major bioactive metabolite with antimicrobial and cytotoxic activity isolated from River Nile derived fungus *Aspergillus awamori*. Beni-Suef Univ. J. Basic Appl. Sci..

[35] Chang M., Wang J., Tian F., Zhang Q., Ye B. (2010). Antibacterial activity of secondary metabolites from *Aspergillus awamori* F12 isolated from rhizospheric soil of *Rhizophora stylosa* Griff. Wei Sheng Wu Xue Bao.

[36] Sabotič J., Bayram E., Ezra D., Gaudêncio S.P., Haznedaroğlu B.Z., Janež N., Ktari L., Luganini A., Mandalakis M., Safarik I. (2024). A guide to the use of bioassays in exploration of natural resources. Biotechnol. Adv..

[37] Kwasny S.M., Opperman T.J. (2010). Static biofilm cultures of Gram-positive pathogens grown in a microtiter format used for antibBiofilm drug discovery. Curr. Protoc. Pharmacol..

[38] Gaudêncio S.P., Bayram E., Lukić Bilela L., Cueto M., Díaz-Marrero A.R., Haznedaroglu B.Z., Jimenez C., Mandalakis M., Pereira F., Reyes F. (2023). Advanced methods for natural products discovery: Bioactivity screening, dereplication, metabolomics profiling, genomic sequencing, databases and informatic tools, and structure elucidation. Mar. Drugs.

[39] Gaudêncio S.P., Pereira F. (2015). Dereplication: Racing to speed up the natural products discovery process. Nat. Prod. Rep..

[40] Ohmomo S., Oguma K., Ohashi T., Abe M. (1978). Isolation of a new indole alkaloid, Roquefortine D, from the cultures of *Penicillium roqueforti*. Agric. Biol. Chem..

[41] Ohmomo S., Sato T., Utagawa T., Abe M. (1975). Isolation of festuclavine and three new indole alkaloids, Roquefortine A, B and C from the cultures of *Penicillium roqueforti*. Agric. Biol. Chem..

[42] Metin B. (2023). *Penicillium roqueforti* secondary metabolites: Biosynthetic pathways, gene clusters, and bioactivities. Fermentation.

[43] Cutler H.G., Crumley F.G., Cox R.H., Wells J.M., Cole R.J. (1984). The biological properties of cyclopenin and cyclopenol. Plant Cell Physiol..

[44] Li J., Wang J., Jiang C.-S., Li G., Guo Y.-W. (2014). (+)-Cyclopenol, a new naturally occurring 7-membered 2,5-dioxopiperazine alkaloid from the fungus *Penicillium sclerotiorum endogenous* with the Chinese mangrove *Bruguiera gymnorrhiza*. J. Asian Nat. Prod. Res..

[45] Cai X.-Y., Wang J.-P., Shu Y., Hu J.-T., Sun C.-T., Cai L., Ding Z.-T. (2022). A new cytotoxic indole alkaloid from the fungus *Penicillium polonicum* TY12. Nat. Prod. Res..

[46] Kuno F., Shiomi K., Otoguro K., Sunazuka T., Omura S. (1996). Arisugacins A and B, novel and selective acetylcholinesterase inhibitors from *Penicillium* sp. FO-4259. II. Structure elucidation. J. Antibiot..

[47] Li H., Jiang J., Liu Z., Lin S., Xia G., Xia X., Ding B., He L., Lu Y., She Z. (2014). Peniphenones A–D from the mangrove fungus *Penicillium dipodomyicola* HN4-3A as inhibitors of *Mycobacterium tuberculosis* phosphatase MptpB. J. Nat. Prod..

[48] Sasaki T., Takagi M., Yaguchi M., Nishiyama K., Yaguchi T., Koyama M. New Antibiotic Substance PF1052 and Its Production. JPH04316578A. Meiji Seika Kaisha Ltd. Published 6 November 1992. https://patents.google.com/patent/JPH04316578A/en.

[49] Gouws L., Botes E., Wiese A.J., Trenkamp S., Torres-Jerez I., Tang Y., Hills P.N., Usadel B., Lloyd J.R., Fernie A. (2012). The plant growth promoting substance, lumichrome, mimics starch and ethylene-associated symbiotic responses in lotus and tomato roots. Front. Plant Sci..

[50] Kawai K., Nozawa K., Nakajima S., Iitaka Y. (1984). Studies on Fungal Products. VII. The Structures of meleagrin and 9-O-p-bromobenzoylmeleagrin. Chem. Pharm. Bull..

[51] Du L., Feng T., Zhao B., Li D., Cai S., Zhu T., Wang F., Xiao X., Gu Q. (2010). Alkaloids from a deep ocean sediment-derived fungus *Penicillium* sp. and their antitumor activities. J. Antibiot..

[52] Gerlach M., Schwelle N., Lerbs W., Luckner M. (1985). Enzymatic synthesis of cyclopeptine intermediates in *Penicillium cyclopium*. Phytochemistry.

[53] Cunningham K.G., Freeman G.G. (1953). The isolation and some chemical properties of viridicatin, a metabolic product of *Penicillium viridicatum* Westling. Biochem. J..

[54] Taniguchi M., Satomura Y. (1970). Isolation of viridicatin from *Penicillium crustosum*, and physiological activity of viridicatin and its 3-carboxymethylene derivative on microorganisms and plants. Agric. Biol. Chem..

[55] Euch I.Z.E., El-Metwally M.M., Frese M., Sewald N., Abdissa N., Shaaban M. (2019). Secondary metabolites from *Penicillium* sp. 8PKH isolated from deteriorated rice straws. Z. Naturforschung C.

[56] Liu Y., Shu Z., Li Y., Chen H., Liu H., Yang X., Liu G., Liu Q. (2023). Deep-sea-derived viridicatol relieves allergic response by suppressing MAPK and JAK-STAT signalling pathways of RBL-2H3 cells. Food Agric. Immunol..

[57] Shu Z., Liu Q., Xing C., Zhang Y., Zhou Y., Zhang J., Liu H., Cao M., Yang X., Liu G. (2020). Viridicatol isolated from deep-sea *Penicillium Griseofulvum* alleviates anaphylaxis and repairs the intestinal barrier in mice by suppressing mast cell activation. Mar. Drugs.

[58] Ma Y.M., Qiao K., Kong Y., Li M.Y., Guo L.X., Miao Z., Fan C. (2017). A new isoquinolone alkaloid from an endophytic fungus R22 of *Nerium indicum*. Nat. Prod. Res..

[59] Moussa A.Y., Albelbisy M.A.K., Singab A.N.B. (2025). The underrepresented quinolinone alkaloids in genera *Penicillium* and *Aspergillus*: Structure, biology, and biosynthetic machinery. Chem. Biodivers..

[60] Lazarovits G., Steele R., Stoessl A. (1988). Dimers of altersolanol-A from *Alternaria solani*. Z. Naturforschung Sect. C-A J. Biosci..

[61] Gu T., Wen Y., Zhou Q., Yuan W., Guo H., Chang W.-L., Yang Q. (2024). Fungal metabolite altersolanol a exhibits potent cytotoxicity against human placental trophoblasts in vitro via mitochondria-mediated apoptosis. Mycotoxin Res..

[62] Yang X., Strobel G., Stierle A., Hess W.M., Lee J., Clardy J. (1994). A fungal endophyte-tree relationship: *Phoma* sp. in *Taxus wallachiana*. Plant Sci..

[63] Galmarini O.L., Stodola F.H., Raper K.B., Fennell D.I. (1962). Fonsecin, a naphthopyrone pigment from a mutant of *Aspergillus fonsecaeus*. Nature.

[64] Leutou A.S., Yun K., Son B.W. (2016). Induced production of 6,9-dibromoflavasperone, a new radical scavenging naphthopyranone in the marine-mudflat-derived fungus *Aspergillus niger*. Arch. Pharmacal Res..

[65] Rho M.-C., Toyoshima M., Hayashi M., Uchida R., Shiomi K., Komiyama K., Omura S. (1998). Enhancement of drug accumulation by andrastin A produced by *Penicillium* sp. FO-3929 in vincristine-resistant KB cells. J. Antibiot..

[66] Rojas-Aedo J.F., Gil-Durán C., Del-Cid A., Valdés N., Álamos P., Vaca I., García-Rico R.O., Levicán G., Tello M., Chávez R. (2017). The biosynthetic gene cluster for Andrastin A in *Penicillium roqueforti*. Front. Microbiol..

[67] Soledade M., Pedras C., Biesenthal C.J. (2001). Isolation, structure determination, and phytotoxicity of unusual dioxopiperazines from the phytopathogenic fungus *Phoma lingam*. Phytochemistry.

[68] Moldes-Anaya A., Rundberget T., Uhlig S., Rise F., Wilkins A.L. (2011). Isolation and structure elucidation of secopenitrem D, an indole alkaloid from *Penicillium crustosum* Thom. Toxicon.

[69] Guimarães D.O., Borges W.S., Vieira N.J., de Oliveira L.F., da Silva C.H.T.P., Lopes N.P., Dias L.G., Durán-Patrón R., Collado I.G., Pupo M.T. (2010). Diketopiperazines produced by endophytic fungi found in association with two *Asteraceae* species. Phytochemistry.

[70] Praseatsook K., Vachiraarunwong A., Sato K., Dissook S., Wanibuchi H., Taya S., Wongpoomchai R., Dejkriengkraikul P., Gi M., Yodkeeree S. (2025). Chemopreventive effects of bioactive peptides derived from Black Soldier fly larvae protein hydrolysates in a rat model of early-stage colorectal carcinogenesis. Int. J. Mol. Sci..

[71] Scholar E., Enna S.J., Bylund D.B. (2007). Ofloxacin. xPharm: The Comprehensive Pharmacology Reference.

[72] Machado Dutra J., Espitia P.J.P., Andrade Batista R. (2021). Formononetin: Biological effects and uses—A review. Food Chem..

[73] Sharma N., Kabra A. (2025). Formononetin: Pharmacological properties and therapeutic potential. Naunyn Schmiedebergs Arch. Pharmacol..

[74] Bjelton L., Wennberg A., Ekman L., Kihlberg R. (1989). N-acetyl-L-tyrosine as a source of tyrosine in intravenous nutrition an experimental study in the rat. Nutr. Res..

[75] Kumar R., Ramachandran U., Raichur S., Chakrabarti R., Jain R. (2007). Synthesis and evaluation of N-acetyl-L-tyrosine based compounds as PPARalpha selective activators. Eur. J. Med. Chem..

[76] Matsumura T., Uryu O., Matsuhisa F., Tajiri K., Matsumoto H., Hayakawa Y. (2020). N-acetyl-l-tyrosine is an intrinsic triggering factor of mitohormesis in stressed animals. EMBO Rep..

[77] Shi Y.-S., Zhang Y., Chen X.-Z., Zhang N., Liu Y.-B. (2015). Metabolites produced by the endophytic fungus *Aspergillus fumigatus* from the stem of *Erythrophloeum fordii* Oliv. Molecules.

[78] Chantarawong W., Kuncharoen N., Tanasupawat S., Chanvorachote P. (2019). Lumichrome inhibits human lung cancer cell growth and induces apoptosis via a p53-dependent mechanism. Nutr. Cancer.

[79] Liu C., Cao Z., Zhang W., Tickner J., Qiu H., Wang C., Chen K., Wang Z., Tan R., Dong S. (2018). Lumichrome inhibits osteoclastogenesis and bone resorption through suppressing RANKL-induced NFAT activation and calcium signaling. J. Cell. Physiol..

[80] Kjer J., Debbab A., Aly A.H., Proksch P. (2010). Methods for isolation of marine-derived endophytic fungi and their bioactive secondary products. Nat. Protoc..

[81] Gözcelioğlu B. (2019). Antioxidant and cytotoxic activity of three Turkish marine-derived fungi. Turk. J. Biochem..

[82] Schoch C.L., Seifert K.A., Huhndorf S., Robert V., Spouge J.L., Levesque C.A., Chen W. (2012). Nuclear ribosomal internal transcribed spacer (ITS) region as a universal DNA barcode marker for Fungi. Proc. Natl. Acad. Sci. USA.

[83] Sun Y., Cai Y., Huse S.M., Knight R., Farmerie W.G., Wang X., Mai V. (2012). A large-scale benchmark study of existing algorithms for taxonomy-independent microbial community analysis. Brief. Bioinform..

[84] Coelho M.P., Suárez-Moo P., Rocha M., Matos A.O.G., Marques V., Margarida S., Mil-Homens M., Prieto-Davó A., Rodrigues C.M.P., Bauermeister A. (2026). Taxonomic diversity and metabolic and pharmacological profiles of marine-derived actinomycetes from the Lisbon and Setúbal Coast, Portugal. Mar. Drugs.

[85] Pinto-Almeida A., Bauermeister A., Luppino L., Grilo I.R., Oliveira J., Sousa J.R., Petras D., Rodrigues C.F., Prieto-Davó A., Tasdemir D. (2022). The diversity, metabolomics profiling, and the pharmacological potential of actinomycetes isolated from the Estremadura Spur pockmarks (Portugal). Mar. Drugs.

[86] Aron A.T., Gentry E.C., McPhail K.L., Nothias L.F., Nothias-Esposito M., Bouslimani A., Petras D., Gauglitz J.M., Sikora N., Vargas F. (2020). Reproducible molecular networking of untargeted mass spectrometry data using GNPS. Nat. Protoc..

